# Spontaneous Nasal Septal Abscess: A Case Report

**DOI:** 10.7759/cureus.58007

**Published:** 2024-04-10

**Authors:** Nur Nadia Abd Rahim, How Kit Thong, Primuharsa Putra Sabir Husin Athar

**Affiliations:** 1 Otorhinolaryngology-Head and Neck Surgery, KPJ Healthcare University, Nilai, MYS; 2 Otorhinolaryngology-Head and Neck Surgery, KPJ Seremban Specialist Hospital, Seremban, MYS

**Keywords:** atraumatic, idiopathic, spontaneous, nasal infection, nasal septal abscess, rhinology, nasal, septal abscess

## Abstract

Nasal septal abscess (NSA) is considered a rhinologic emergency. Fortunately, the incidence of NSA has markedly reduced due to the introduction of antibiotics and easy access to medical care. NSA commonly results from infection in the space between the nasal septum and the overlying mucoperichondrium and/or mucoperiosteum, typically secondary to nasal septal hematoma, but it can also be idiopathic. Prompt diagnosis and intervention are critical to avoid further complications. This paper reports the case of a 46-year-old man with no known risk factors for NSA. He was treated with broad-spectrum antibiotics, and the surgical treatment involved incision and drainage with the intraoperative placement of a Penrose drain and a silastic sheet on postoperative day five. The patient was discharged without complications such as septal perforation or saddle nose deformity.

## Introduction

Nasal septal abscess (NSA) is a localized accumulation of pus that forms between the bony or cartilaginous structure of the nasal septum and the surrounding mucoperichondrium or mucoperiosteum. This condition can cause significant discomfort and health issues. A classification system proposed by Beck AL et al. categorizes the etiology of nasal septal abscesses into three distinct groups: primary causes (such as trauma), secondary causes, which usually stem from infections, and cases with an idiopathic origin, where the exact cause remains unclear [1]. There have also been cases of abscesses in the maxillofacial region, including the nasal septum, originating from the odontogenic region, albeit occurring infrequently [2]. While spontaneous cases of septal abscess are infrequently documented in medical literature, the overall prevalence of NSA is notable within the pediatric population and up to 15% in nasal trauma cases. A recent study of 36 patients found that up to 17% of cases had no etiologic factors found, with diabetes mellitus being the most common cause of NSA and Staphylococcus aureus the most common organism cultured [3]. This condition often presents with a cluster of characteristic symptoms, including progressive nasal congestion, localized nasal pain, fever, and headache [4]. These symptoms can collectively contribute to a diminished quality of life for affected individuals, underscoring the importance of timely diagnosis and appropriate management. This case is reported to alert healthcare personnel to the possibility of a patient with no known comorbidities developing a spontaneous nasal abscess.

## Case presentation

**Figure 1:**
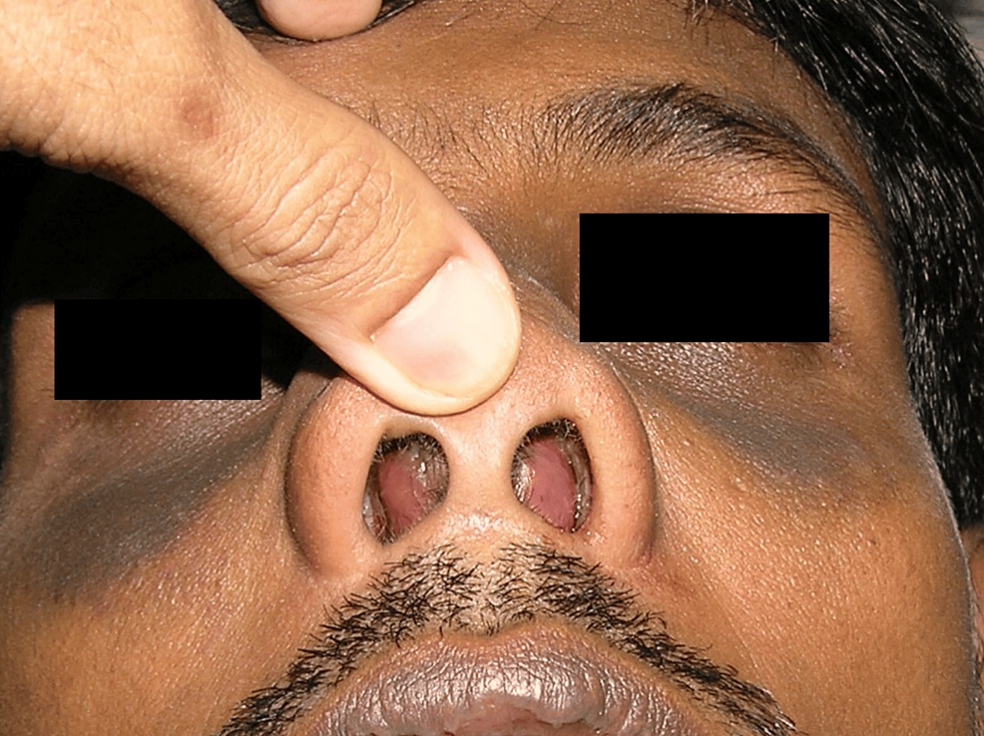
A picture showing bilateral septal swelling.

A 46-year-old man with no known comorbidities was referred to our Otorhinolaryngology - Head and Neck Surgery Clinic in Nilai, Malaysia, by a local clinic with a history of nasal tip pain for the past five days. He also had severe progressive bilateral nasal blockage and upper lip swelling. However, there was no associated fever, blurred vision, headache, rhinorrhea, or previous history of surgery or trauma. He appeared comfortable and exhibited normal vital signs. His vital signs on arrival were stable, with a blood pressure of 125/74 mmHg, a pulse rate of 80 beats per minute, a body temperature of 37 degrees Celsius, and a normal blood glucose level of 5.0 mmol/L. Physical examination revealed no neurological deficit, and inspection of the nose revealed a swollen and tender nasal tip region. There was no presence of cervical lymphadenopathy in this patient. Anterior rhinoscopy showed bilateral nasal obstruction, as evidenced in Figure 1. Nasoendoscopy showed severe bilateral nasal obstruction caused by a bilateral anterior septum bulge, which was tender and fluctuant on palpation. Oral examination revealed no dental caries or other abnormalities in the oral cavity.

**Figure 2:**
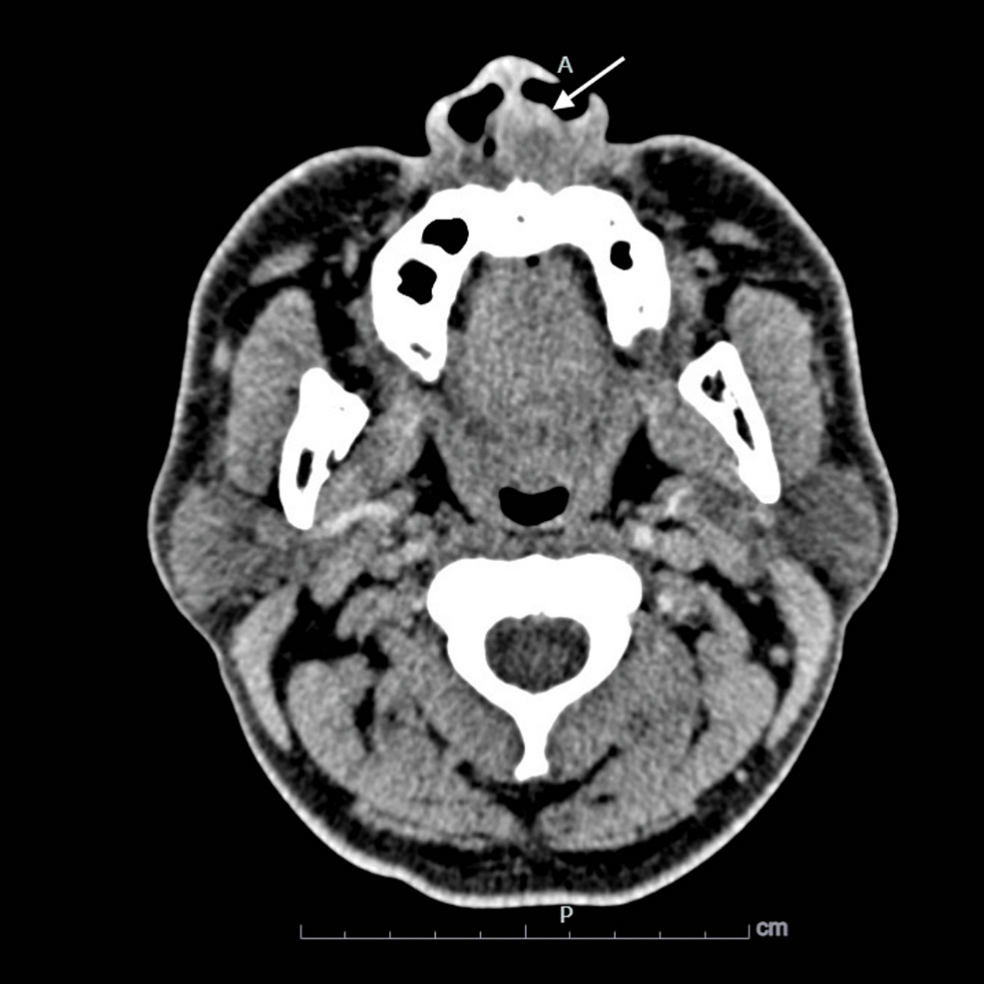
Computed tomography scan of the paranasal sinuses and post-nasal space, showing a lesion with a thickened, enhancing wall and a hypodense area at the anterior aspect of the nasal septum. This lesion, featuring erosion and right deviation of the nasal septum, is consistent with an early-forming abscess.

Relevant laboratory findings included a raised total white blood cell count of 22 x 10^3/uL (age-appropriate, normal range for males: 4.0-10.3 x 10^3/uL) with a neutrophil predominance of 87.7% (age-appropriate, normal range for males: 37.2-70%), and the random blood glucose level was within the normal limit, at 5.7 mmol/L (age-appropriate, normal range for both sexes: 4.1-6.1 mmol/L). A contrast-enhanced computed tomography scan of the paranasal sinuses and post-nasal space, as shown in Figure 2, revealed a thickened wall enhancing lesion with a hypodense area measuring 4.3 x 3.3 cm at the anterior aspect of the nasal septum, causing obstruction to the nasal cavity bilaterally and an erosion to the nasal septum with right deviation. The patient was admitted and promptly started on both empirical IV ceftriaxone 1 gram once daily and metronidazole 500 mg thrice daily dosing to cover any odontogenic origin of infection for five days [5].

Incision and drainage of the septal swelling were performed under general anesthesia, and no local anesthetic was used as it was a case of a NSA a few hours after admission. A unilateral vertical (Killian) incision was made at the bulging and fluctuant area at the left anterior septal region. 5 cc of frank pus was drained from both sides of the septum, and hemostasis was secured with the temporary application of a topical adrenaline 1:1000 solution with a half-inch ribbon gauze. The abscess cavity was then flushed with a diluted povidone 0.5% solution. Presumably, there was a communication caused by nasal septal necrosis; however, the surgeon was unable to inspect the full length of the septum due to the limited surgical field and the underlying unhealthy tissue occupying the abscess cavity. A small corrugated drain was inserted and sutured to the septum to maintain patency. The pus sample was sent for culture and sensitivity testing.

A septal Silastic sheet was placed and sutured under local anesthesia as an office procedure on postoperative day 5 to obliterate the potential septal space. The Silastic sheet was inserted bilaterally and anchored through the septum to each other, acting as a compressive dressing in a sandwich effect. Pus culture revealed mild growth of Staphylococcus aureus sensitive to Ceftriaxone; hence, the antibiotic was continued for five more days before the patient's discharge. The Silastic sheet was removed three weeks after surgery. Follow-up visit examination showed complete abscess resolution without septal perforation or saddle nose deformity.

## Discussion

NSA is a rare condition, as evidenced by a report of only 6 cases within a 10-year study in a hospital in Taipei [6]. The most common symptoms are nasal pain and nasal obstruction [3]. Some cases may also present with submandibular lymph node enlargement, as it correlates with the drainage passage of the lymphatic system for the nasal septum region [7]; however, it was not observed in this patient. The most common etiologies include facial trauma, nasal cavity or sinus infections, and iatrogenic causes. Another possible etiology of NSA is infection by anaerobic bacteria from the oral flora, post-upper alveolus injury, as reported in a case study [8]. There are reports of NSAs in individuals with immunosuppression, which have been attributed to factors such as insulin and non-insulin-dependent diabetes [9], HIV [10], hematological malignancies and chemotherapy [11], and end-stage kidney disease [12]. Interestingly, during the COVID-19 pandemic, one report described a patient with beta-thalassemia major developing a NSA post-nasal swab test for COVID-19 [13]. Spontaneous nasal septal hematoma or abscess without any identifiable cause, as illustrated by the case being discussed, is rare.

Rapid diagnosis, incision, and drainage of the abscess, coupled with the administration of intravenous antibiotics, are crucial for such cases [5]. This patient presented on day 5 of symptoms, well before the average onset of complications in NSA cases, which is day 8 [5]. The absence of cervical lymphadenopathy correlates with a study in Bulgaria, which reported only 5.71% of patients with maxillofacial region diseases presented with this symptom [14]. Most patients with NSA receive immediate management with IV broad-spectrum antibiotics, for example, amoxicillin/clavulanic acid, penicillin, and quinolone [5], as the location of the affected area is in the danger triangle of the face, which puts the patient at risk for cavernous sinus thrombosis, a rapid and fatal condition [15]. Antibiotic choice is re-evaluated after the results of culture and sensitivity tests. Studies have reported high incidences of Staphylococcus aureus growth in the aspirated septal samples, as high as 71.4% [5]. Patients at higher risk for methicillin-resistant Staphylococcus aureus (MRSA) due to advanced age, recent use of antibiotics or hospital admission, healthcare workers, or HIV patients should receive antibiotics for MRSA [6]. This patient received parenteral broad-spectrum antibiotics, based on the sensitivity to Staphylococcus aureus from culture.

Proposed surgical management generally includes emergency incision and drainage under general anesthesia, irrigation of the nasal cavity using 0.9% saline, optional placement of a Penrose drain, and nasal packing to prevent and reduce the reaccumulation of fluid [3, 5, 16]. In this case, a small corrugated drain was inserted through an incision site for drainage. The drain was kept for five days. Early reconstruction of the septal cartilage is necessary in cases of septal damage to prevent saddle nose deformity and to yield an exceptional functional and cosmetic result, especially in the pediatric and adolescent population [17].

Complications reported from NSA can be categorized as local and systemic. Local complications include saddle nose deformity [18] and naso-oral fistula; systemic complications include meningitis, subdural abscess, septic arthritis, sigmoid sinus thrombosis, and skull base osteomyelitis [6].

In view of the negative history and investigation results for risk factors that may predispose this patient to NSA, the classification of this case as spontaneous NSA is deemed appropriate. This patient was lost to follow-up; therefore, we could not assess long-term complications following surgery.

## Conclusions

Both GPs and otolaryngologists are advised to maintain constant vigilance and a heightened index of suspicion to prevent potential morbidities associated with this condition. NSA primarily relies on clinical diagnosis, necessitating the prompt initiation of treatment. The absence of risk factors for NSA, coupled with the unavailability of imaging investigations, should not impede the initiation of treatment. This is crucial, as the numerous complications that may arise range from saddle nose deformity to the more severe cavernous sinus thrombosis, due to the location of the affected area being in the danger triangle of the face.
